# Supplementary material for: Phloroglucinol Derivative Carbomer Hydrogel Accelerates MRSA-Infected Wounds’ Healing
Source: Int J Mol Sci. 2022 Aug 4;23(15):8682. doi: 10.3390/ijms23158682 (PMC9369305; doi:10.3390/ijms23158682)
Supplement: Supplementary file 1 [file ijms-23-08682-s001.zip › ijms-1845224-supplementary.pdf]

Supporting Information

Phloroglucinol Derivative Carbomer Hydrogel Accelerates MRSA-infected Wounds' Healing

Author: Xiaosu Huang[1,] Junhua Yang[2], Renyue Zhang[3], Lianbao Ye[3], Ming Li[4*#], Weiqiang Chen[1,5*#]

[1]School of Nursing, Guangdong Pharmaceutical University, Guangdong, China; 15915736152@163.com (X, H)

[2]Department of Anatomy, School of Biosciences & Biopharmaceutics, Guangdong Pharmaceutical University, Guangzhou, Guangdong, China; jhyang2018@gdpu.edu.cn (J.Y)

[3]School of Pharmacy, Guangdong Pharmaceutical University, Guangdong, China; z1589557607@163.com (R.Z); yelb7909@163.com (L.Y)

[4]School of Life Science and Biopharmaceutics, Guangdong Pharmaceutical University, Guangdong, China; lim99011@gdpu.edu.cn (M.L)

[5]Guangdong Key Laboratory of Pharmaceutical Bioactive Substances, Guangdong Pharmaceutical University, Guangdong, China; wqc@gdpu.edu.cn (W.C)

[#] Represents co-corresponding author

*Correspondence: Ming Li, mail: lim99011@gdpu.edu.cn; Weiqiang Chen, Tel: +86-020-34055529, mail: wqc@gdpu.edu.cn.

Supplenmentary method

1. Antimicrobial susceptibility testing. Well diffusion method:

Methicillin-Resistant Staphylococcus aureus (MRSA) was obtained from Guangdong Key Laboratory of Pharmaceutical Bioactive Substances, (Guangdong Pharmaceutical University, Guangzhou, China). Antimicrobial activity of PD using the agar well diffusion method as recommended by Clinical and Laboratory Standards Institute (CLSI) guidelines [1]. The turbidity of the bacterial suspension was checked against a 0.5 McFarland turbidity standard to obtain a bacterial cell count of approximately 1-3×10^8 CFU/mL per bacterium tested. Vancomycin (500 μg/mL) was used as standard antimicrobial drug. Application of 50 μL of LMRSA on nutrient agar with a spreading stick. Then, the sterile drug paper sheets were applied on agar to observed for

antimicrobial activity. After incubation at 37 °C for 24 hours, the results were noted by measure of the size of the zone of inhibition. Inhibition circle results are indicated as mean ± standard deviation. By pharmacological test methods: circle of inhibition < 6 mm, drug sensitivity is determined as -; between 6 and 10 mm, as +; between 10 and 16 mm, drug sensitivity as ++; 16 and 26 mm, as +++; greater than 26 mm, as ++++.

Supplenmentary results

1. Structure of PD was shown in Fig. S1.

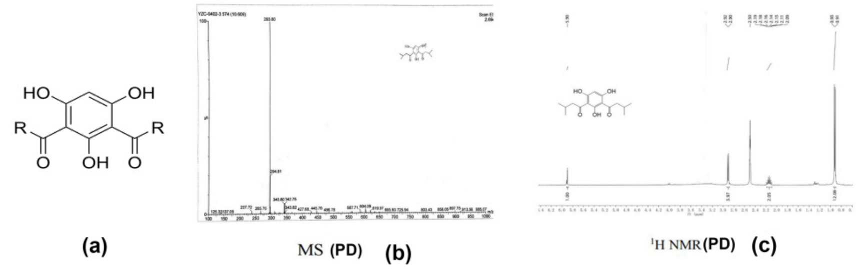

Figure S1: (a) The Structure, (b) mass spectra, and (c) hydrogen spectrum of the phloroglucinol derivative.

2. In vitro antibacterial activity of PD against MRSA.

As shown in Fig. S2, compared to DMSO group, the circle of inhibition tfor PD, Van was 16.34±0.24 mm (+++) and 15.34 ± 0.36 mm (++) (*P*< 0.01), respectively. In addition, PD showed better anti-MRSA activity than vancomycin.

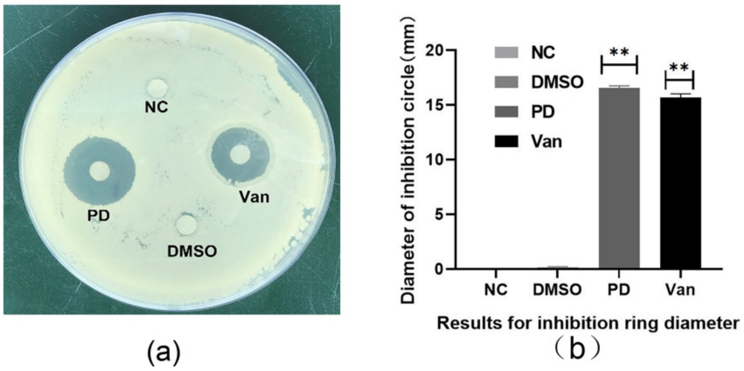

Figure S2: In vitro antibacterial activity of PD against MRSA. (a) Representative photograph of the zone of inhibition. (b) Antimicrobial activities of PD and Van at concentration 500 μg/ml against MRSA (** p <0.01, compared to NC group).

Abbreviations: PD: Phloroglucinol derivative; Van: Vancomycin; NC: Negative control.

References

1. Moglad EH. *Loranthus acaciae*: Alternative medicine for β-lactamase producer

and methicillin-resistant *Staphylococcus aureus*. Saudi J Biol Sci 2021; 28: 1835-1839. doi: 10.1016/j.sjbs.2020.12.029.
